# The effectiveness of different acupuncture therapies for neck pain: A protocol for systematic review and/or network meta-analysis

**DOI:** 10.1097/MD.0000000000025379

**Published:** 2021-04-23

**Authors:** Hyo-Rim Jo, Eun-Ji Noh, Se-Hee Oh, Seong-Kyeong Choi, Won-Suk Sung, Su-Ji Choi, Dong-Il Kim, Seung-Ug Hong, Eun-Jung Kim

**Affiliations:** a Department of Acupuncture & Moxibustion; b Department of Obstetrics & Gynecology, College of Korean Medicine, Dongguk University Graduate School; c Department of Ophthalmology, Otolaryngology and Dermatology, Dongguk University Ilsan Oriental Hospital, Gyeongi-do; d Department of Acupuncture & Moxibustion, Dongguk University Bundang Oriental Hospital, Seongnam-si; e Department of Obstetrics & Gynecology, Dongguk University Ilsan Oriental Hospital, Gyeonggi-do; f Department of Acupuncture & Moxibustion, Dongguk University, Seoul, Republic of Korea.

**Keywords:** acupuncture, neck pain, network meta-analysis, protocol, systematic review

## Abstract

**Background:**

Neck pain is common musculoskeletal disorders in adult population. Acupuncture treatment has been widely used for treating neck pain. Nevertheless, previous systematic reviews (SRs) on acupuncture for neck pain remain controversial, and there is no SR for the comparative efficacy and safety of various types of acupuncture. Therefore, this study aims to evaluate and rank the effectiveness and safety of different types of acupuncture for neck pain by SR and network meta-analysis.

**Methods:**

Nine databases will be searched, including Ovid-MEDLINE, EMBASE, Cochrane library, China National Knowledge Infrastructure (CNKI), KoreaMed, Korean medical database (KMBASE), Korean Studies Information Service System (KISS), ScienceON, and Oriental Medicine Advanced Searching Integrated System (OASIS) from their inception to July 2021. The primary outcome is the change of pain intensity. A frequentist network meta-analysis will be performed to compare all relative outcomes of different acupuncture methods, using R software. The quality of included randomized controlled trials will be assessed by Cochrane Collaboration “risk of bias” tools and the evidence will be evaluated by the Grading of Recommendations Assessment, Development and Evaluation instrument.

**Results:**

The final findings of this network meta-analysis will be published in a recognized journal.

**Conclusions:**

Our study will evaluate and compare the effectiveness of various types of acupuncture for neck pain and provide clinicians with best option for what types of acupuncture treatments are effective.

**Trial registration number:**

INPLASY202120041

## 1 Introduction

Neck pain is one of the common musculoskeletal disorders in adult population. According to 2018 health statistics—National Health Interview Survey, the incidence of neck pain accounts for 15.7% for adults.^[1]^ Some studies suggested that a high prevalence of neck pain is associated with women, performing occupational activities while sitting and leaning, low levels of education and income, and suffering from ≥2 diseases.^[2,3]^

A high proportion of the neck pain becomes recurrent or chronic. Most people with neck pain do not experience complete symptom relief, and recur within 1 to 5 years in 50% to 85% of patients with neck pain. One study reported that 30% of patients show chronic progress and the problems persist for at least 12 months in 37% of patients with neck pain.^[4]^ It affects the physical, social, and psychological well-being of individuals, and increases the costs in health care expenditure. According to Global Burden of Disease 2010 study of 291 conditions, neck pain ranked 21st in terms of overall burden and 4th in terms of overall disability.^[5]^

Although conventional treatments such as medication, injection, or surgery have a therapeutic effect on neck pain,^[6–9]^ some of these treatments have poor evidence, and even had some serious adverse effects including renal dysfunction, hemorrhagic gastritis/ulcer, myocardial infarction, spinal cord and nerve injury, epidural hematoma.^[10–12]^ For these reasons, many patients divert their attention to other treatments, such as complementary and alternative medicine (CAM).

As one of CAM therapies, acupuncture treatment has analgesic effects on musculoskeletal pain through neurophysiological and neurochemical mechanisms. Acupuncture can mediate short term analgesia by activating endogenous antinociceptive systems and descending inhibitory systems.^[13]^ Acupuncture-induced analgesia also can be associated with biopsychosocial pain management system in prefrontal cortex. The dual, opposing roles of medial prefrontal cortex mediate pain chronification or inhibition of pain perception so these opposite interactions should be considered for pain control.^[14]^

Acupuncture is frequently accepted and recommended for neck pain in Korea.^[15]^ Recently, while many randomized controlled trials (RCTs) have conducted various types of acupuncture treatment for cervical pain,^[16–18]^ the systematic reviews (SRs) have shown the effectiveness of acupuncture to be controversy. Several SRs have reported that acupuncture improves pain and disability associated with neck pain superior to sham acupuncture or no treatment but similar to active control group such as medications, massage, and physical therapy.^[19,20]^ Furthermore, Smith et al^[21]^ suggested it is no more effective than placebo, and some SRs^[19,22,23]^ reported inconsistent comparative effects between acupuncture and other interventions. These reviews included various types of acupuncture treatments, and due to the its diversity, the relative effects of each individual option have not yet been studied and compared.

Therefore, we will conduct SR and network meta-analysis to evaluate the effectiveness on neck pain and to estimate comparative effectiveness of different types of acupuncture.

## 2 Methods

This protocol will be reported according to the Preferred Reporting Items for Systematic Reviews and Meta-Analysis Protocols (PRISMA-P) Statement.^[24]^ The research protocol has been registered on INPLASY (Registration number: INPLASY202120041). No ethical statement is required as this study does not require patient recruitment and personal data collection.

### 2.1 Eligibility criteria

#### 2.1.1 Type of participants

Adult patients (>18 years) who had cervical pain or cervical intervertebral disc herniation with or without radicular symptoms will be included. We will exclude patients who have specific reasons for neck pain, including whiplash injuries, athletic injuries. There will be no restriction on sex, ethnicity, duration of illness, or disease severity.

#### 2.1.2 Type of interventions and comparators

We will include a single use of acupuncture to compare the effects of various types of acupuncture treatments. Interventions in the treatment group will include manual acupuncture, electroacupuncture, warm acupuncture, fire acupuncture, and acupuncture point thread-embedding. Control interventions will include sham acupuncture, usual care, western medication, no treatment, and one of the aforementioned acupuncture treatments.

#### 2.1.3 Type of outcomes

The primary outcome measure is the change of pain intensity, as measured by Visual Analogue Scale, Neck Pain Questionnaire, or other validated assessment tools. Secondary outcome measures are to be considered depending on review findings, such as functional status (effective rate, curative rate, and range of motion), disability (Neck Disability Index), and adverse events.

#### 2.1.4 Type of studies

This study will include all relevant RCTs using various types of acupuncture therapies for neck pain and the first period in randomized cross-over trials. Non-RCT studies including care studies, quasi-RCT, experimental studies, cohort studies, and RCTs published in the form of letters-to-the-editor, and conference abstracts will be excluded.

### 2.2 Information sources and search strategy

The following electronic databases will be searched from their inception to July 2021: Ovid-MEDLINE, EMBASE, Cochrane library, China National Knowledge Infrastructure (CNKI), KoreaMed, Korean medical database (KMBASE), Korean Studies Information Service System (KISS), ScienceON, and Oriental Medicine Advanced Searching Integrated System (OASIS).

Researchers will search using medical search headings (MeSH) terms and key words with the limitation of Chinese, English, and Korean language. The following search terms will be used in each database's own language: researchers will perform search using terms with combination of neck pain related terms (such as neck pain, cervical pain, cervicodynia, cervicalgia, cervical intervertebral disc displacement/degeneration, cervical spondylosis) and treatments (the names of various acupuncture types, such as acupuncture, electroacupuncture, warm acupuncture, fire acupuncture, thread-embedding). If needed, a manual search, such as the textbooks on acupuncture and the references of the retrieved SRs, will be also done (Table 1).

**Table 1:** Search strategy in MEDILINE (Ovid SP).

No.	Search terms
1	Neck pain/or thoracic outlet syndrome/or cervical rib syndrome/or Torticollis/
2	(“cervical pain” or “neckache” or “cervicodynia” or “cervicalgia” or “brachialgia” or “brachial neuritis” or “brachial neuralgia” or “neck pain” or neck injur^∗^ or brachial plexus neuropath^∗^ or “brachial plexus neuritis”).mp.
3	exp Brachial Plexus Neuropathies/ or exp brachial plexus neuropathies/ or exp brachial plexus neuritis/
4	(cervico brachial neuralgia or cervicobrachial neuralgia).ti,ab.
5	(monoradicul^∗^ or monoradicl^∗^).tw.
6	Or 1 to 5
7	neck/ or neck muscles/ or atlanto-axial joint/ or atlanto-occipital joint/ or Cervical Atlas/ or spinal nerve roots/ or axis/ or odontoid process/ or Thoracic Vertebrae/
8	exp cervical plexus/ or exp cervical vertebrae/ or exp brachial plexus/
9	(odontoid^∗^ or cervical or occip^∗^ or atlant^∗^).tw.
10	(“cervical vertebrae” or “cervical plexus” or “cervical spine” or “neck” or “trapezius” or (neck adj3 muscles) or (brachial adj3 plexus) or (thoracic adj3 vertebrae) or (thoracic adj3 spine) or (thoracic adj3 outlet)).mp.
11	Or 7 to 10
12	exp pain/ or exp injuries/
13	(“pain” or “ache” or “sore” or “stiff” or “discomfort” or injur^∗^ or neuropath^∗^).mp.
14	12 or 13
15	11 and 14
16	Radiculopathy/ or myofascial pain syndromes/ or Polyradiculopathy/ or Fibromyalgia/ or spondylitis/ or discitis/ or spondylosis/ or spondylolysis/ or spondylolisthesis/
17	exp temporomandibular joint disorders/ or exp temporomandibular joint dysfunction syndrome/ or exp “Sprains and Strains”/ or exp Spinal Osteophytosis/ or exp Neuritis/ or exp Arthritis/
18	(“radiculopathy” or “radiculitis” or “temporomandibular” or myofascial pain syndrome^∗^ or thoracic outlet syndrome^∗^ or “spinal osteophytosis” or “neuritis” or “spondylosis” or “spondylitis” or “spondylolisthesis”).mp.
19	Or 16 to 18
20	11 and 19
21	exp neck/ or exp cervical vertebrae/
22	Thoracic Vertebrae/
23	(“neck” or “cervical spine” or (thoracic adj3 vertebrae) or (thoracic adj3 spine)).mp.
24	Or 21 or 23
25	Intervertebral Disk/
26	((disc or discs) or (disk or disks)).mp.
27	25 or 26
28	24 and 27
29	(herniat^∗^ or prolapsed^∗^ or displace^∗^ or degenerate^∗^ or “slipped” or (bulge or bulged or bulging)).mp.
30	28 and 29
31	intervertebral disk degeneration/ or intervertebral disk displacement/
32	(“intervertebral disc displacement” or “intervertebral disc degeneration” or “intervertebral disc degeneration”).mp.
33	31 or 32
34	24 and 33
35	6 or 15 or 20 or 30 or 34
36	(“acupuncture” or “acu-puncture” or “needling” or “acupressure” or “electric acupuncture” or “electroacupuncture” or “warm needle” or “warming acupuncture” or “warmed electroacupuncture” or “warm acupuncture” or “fire needle” or “fire acupuncture” or “catgut implantation” or catgut emb^∗^ or thread emb^∗^).tw.
37	exp Acupuncture Points/ or exp Acupuncture Therapy/ or exp Acupuncture/ or exp Electroacupuncture/ or exp Catgut/
38	36 or 37
39	35 and 38
40	(randomized controlled trial or controlled clinical trial).pt.
41	(randomized or placebo or randomly or trial or groups).ab.
42	drug therapy.fs.
43	Or 40 to 42
44	exp animals/ not humans.sh.
45	43 not 44
46	39 and 45

### 2.3 Literature selection and data extraction

**Figure 1:**
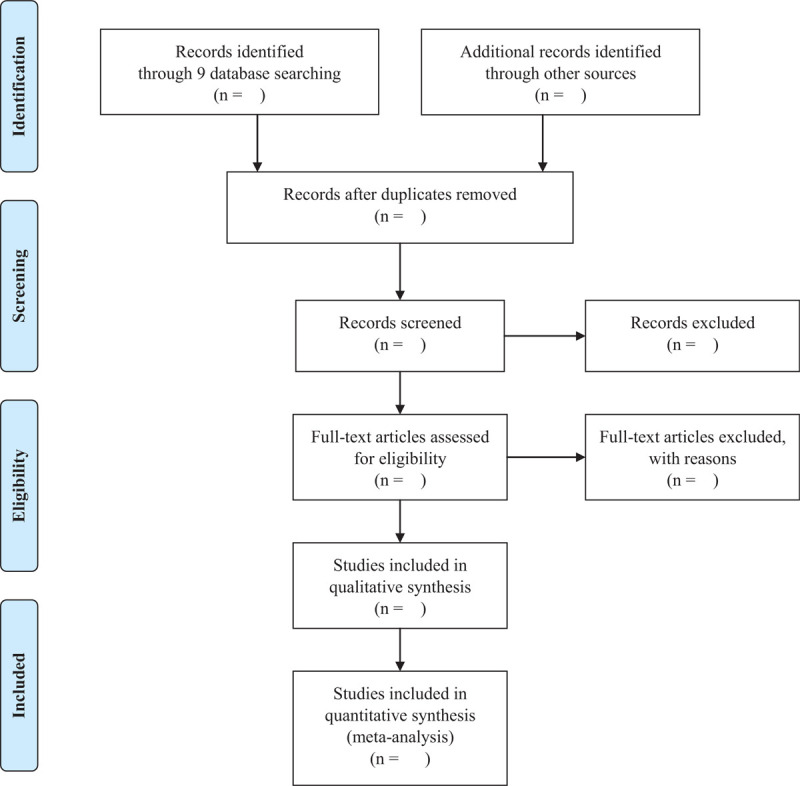
PRISMA flow diagram. PRISMA = Preferred Reporting Items for Systematic Reviews and Meta-Analyses.

All searched studies will be imported into Endnote X20. Two independent researchers will screen the output based on the titles, abstracts, and full-text (if needed) to exclude the duplicates and irrelevant articles. After then, the 2 researchers will review the studies by reading the full-texts. Data extraction will be performed independently by 2 authors and included the following data: study characteristics (author and publication year), sample size, age range, sex, intervention, comparator, treatment frequency, duration, outcomes, results, and adverse events from each of the included RCTs. If necessary, we will try to contact the original author for missing data or clarification for unclear information. If there are any discrepancies, 2 reviewers will solve the problem through discussion. If the disagreement persists, a third reviewer will make final decision (Fig. 1).

### 2.4 Quality assessment

The “Risk of Bias (RoB) Tool” of Cochrane Collaboration will be used to assess the risk of bias. In 7 domains (sequence generation, allocation concealment, blinding of participants, blinding of outcome assessors, incomplete outcome data, selective outcome reporting, and other bias), the assessment result will be divided into “low risk,” “high risk,” and “unclear” by independent 2 reviewers. If there is a disagreement between 2 researchers, the final evaluation will be conducted by a third researcher.^[25]^

### 2.5 Statistical analysis

We will adopt the random-effects model for pairwise meta-analyses of direct evidence, while a random-effects network meta-analysis within a frequentist framework will be performed. A different measure used for evaluating the same outcomes will be analyzed by calculating the dichotomous data as Mantel-Haenszel odds ratios (ORs) and the continuous outcomes as standardized mean difference (SMD). We will present 95% confidence intervals for all outcomes.

We will use R software (http://www.r-project.org/, version 4.0.3) for network meta-analysis to compare direct and indirect evidence. The numerical variables will be presented as the ORs and SMD with 95% credible intervals. The rank of treatments for each outcome will be conducted as surface under the cumulative ranking curve (SUCRA), where SUCRA values indicate the efficacy possibility of the intervention.^[26]^ If the available data are not suitable for synthesis, we will conduct a narrative review and summarize the evidences.

### 2.6 Heterogeneity test

Before the combination of effect size, we will examine the potential effects of heterogeneity on the effect estimates. The heterogeneity assessment will be estimated by *I*^2^. When *I*^2^ > 50%, there is heterogeneity between studies and the source of heterogeneity need to be further searched. When *I*^2^ < 50%, it is considered to be less heterogeneity or no obvious heterogeneity.

### 2.7 Consistency assessment

We will evaluate the presence of local inconsistency and global inconsistency between direct and indirect evidence. If there is no significant difference (*P* > .05) between direct and indirect comparisons, a consistency model is adopted, and stability of the results is verified by the inconsistency model.

### 2.8 Subgroup analysis

If possible, the subgroup analysis will be conducted based on age, sex, and duration of disease and sensitivity analysis will be performed additionally.

### 2.9 Small sample effect/publication bias

If >10 studies are included in the network meta-analysis, a funnel plot is planned to evaluate the presence of small sample effects or publication bias.

### 2.10 Sensitivity analysis

If necessary, a sensitivity analysis will be conducted by the exclusion method that eliminates effects of trials with small sample size and removes studies rated as high risk of bias. When each of these studies is excluded, the remaining studies will be reanalyzed to verify the stability of the results. If the qualitative change in the combined effect is not observed, it can be indicated that the results are stable.

### 2.11 Evaluating the quality of the evidence

The Grading of Recommendations Assessment, Development and Evaluation (GRADE) method will also be used to grade the quality of evidence for each outcome.^[27]^ The GRADE rates evidence quality into “high,” “medium,” “low,” and “very low” on the basis of risk of bias, inconsistency, imprecision, indirection, and publication bias.

## 3 Discussion

Neck pain is a common musculoskeletal disorder that causes morbidity and disability in daily life and at work. Conventional treatments and surgical methods are helpful but have some limitations. Acupuncture has been widely used for musculoskeletal pain, but inconsistent results have been reported across SRs. In addition, there is no SR for the relative effect of acupuncture in various types, thus lacking clear guideline for clinicians. Therefore, we will use network meta-analysis to compare and rank the effectiveness of different types of acupuncture therapies and assess the quality of evidence with the GRADE approach. We hope that our results will provide clinicians with the best options and help lower uncertainty about the effectiveness of acupuncture treatment for treating neck pain.

## Author contributions

**Conceptualization:** Hyo-Rim Jo, Eun-Ji Noh, Se-Hee Oh.

**Data curation:** Su-Ji Choi.

**Formal analysis:** Eun-Ji Noh, Seung-Ug Hong.

**Funding acquisition:** Eun-Jung Kim.

**Methodology:** Hyo-Rim Jo, Eun-Ji Noh, Se-Hee Oh.

**Project administration:** Dong-Il Kim, Seung-Ug Hong.

**Supervision:** Eun-Jung Kim.

**Writing – original draft:** Hyo-Rim Jo, Se-Hee Oh.

**Writing – review & editing:** Seong-Kyeong Choi, Won-Suk Sung, Eun-Jung Kim
